# Density-Dependent Compensatory Growth in Brown Trout (*Salmo trutta*) in Nature

**DOI:** 10.1371/journal.pone.0063287

**Published:** 2013-05-03

**Authors:** L. Fredrik Sundström, Rasmus Kaspersson, Joacim Näslund, Jörgen I. Johnsson

**Affiliations:** University of Gothenburg, Department of Biological and Environmental Sciences, Göteborg, Sweden; Swansea University, United Kingdom

## Abstract

Density-dependence is a major ecological mechanism that is known to limit individual growth. To examine if compensatory growth (unusually rapid growth following a period of imposed slow growth) in nature is density-dependent, one-year-old brown trout (*Salmo trutta* L.) were first starved in the laboratory, and then released back into their natural stream, either at natural or at experimentally increased population density. The experimental trout were captured three times over a one-year period. We found no differences in growth, within the first month after release (May-June), between the starved fish and the control group (i.e. no evidence of compensation). During the summer however (July-September), the starved fish grew more than the control group (i.e. compensation), and the starved fish released into the stream at a higher density, grew less than those released at a natural density, both in terms of weight and length (i.e. density-dependent compensation). Over the winter (October-April), there were no effects of either starvation or density on weight and length growth. After the winter, starved fish released at either density had caught up with control fish in body size, but recapture rates (proxy for survival) did not indicate any costs of compensation. Our results suggest that compensatory growth in nature can be density-dependent. Thus, this is the first study to demonstrate the presence of ecological restrictions on the compensatory growth response in free-ranging animals.

## Introduction

Individual growth rates are determined by trade-offs between benefits and immediate and/or delayed costs affecting fitness through effects on behaviour, physiology, morphology, reproduction, and longevity [1]–[3]. Ecological factors that affect growth rate include food availability and quality, predation risk, competition from conspecifics, photoperiod, and temperature [4]–[6]. Several of these factors act in a density-dependent way so that, for example, when the number of conspecifics increases, individual growth rate decreases [7]–[10]. At the same time, many organisms can buffer against environmental variation by adjusting behavior and/or physiology to stabilize their growth trajectory [2], [11], [12].

One particular response that has been observed in many animal taxa is compensatory growth, which is a period of unusually rapid growth following a period of depressed growth [2], [3], [13]–[15]. The increased growth during the compensatory period is commonly achieved through hyperphagia, i.e. an increase in food intake, but it can also arise from a reduced metabolic rate and/or improved conversion efficiency [13], [14], [16]. Observations of compensatory growth under natural conditions [17]–[19], suggests that some of the factors apparently restricting normal growth can be circumvented during compensation. However, the extent to which compensatory growth is regulated by the same ecological factors as normal growth is not known. For example, in the laboratory, roach (*Rutilus rutilus*) do not normally grow at temperatures below 12°C, but will do so if the growth is compensatory [20] showing that the behavioral decision to feed and physiological regulation of growth may be different for normal growth compared to compensatory growth.

In the present study we investigated whether compensatory growth is affected by population density, an important ecological factor that has been shown experimentally to reduce growth under normal growth conditions (i.e. non-compensatory) in nature [7]. Hence, we combine the observation that animals are able to compensate at natural density (i.e. the animal can “at will” grow faster than normal) with the observation that growth is density-dependent (i.e. when individuals compete for space and/or food). Because the faster compensatory growth can be observed under normal growth conditions when we know that growth is density-dependent, compensatory growth may be regulated by mechanisms that are different from those acting during normal growth.

To examine whether density-dependence operates on compensatory growth, we carried out a study on a natural population of one-year-old brown trout. First, we deprived trout of food in the laboratory and then returned them to a stream subsection with a natural density (i.e. density at the time of release) or experimentally increased density of conspecifics and monitored their growth, survival, and movement over a year.

## Methods

### Study site and species

The study was conducted in a 700 m experimental section of the small coastal stream Norumsån in south-western Sweden from the spring of 2008 until the spring of 2009. The stream has a width of <1–5 meters and a depth ranging from a few centimetres in shallow habitats at low flow rates to more than a meter in deeper pools. It runs through deciduous forest with much vegetative overhang. The dominant fish species is sea-migratory brown trout (>95% of all individual fish), which spend about two years in the stream before migrating to the sea. Larger trout typically migrate sooner than smaller and size appears to be the main determinant of migration [21], [22]. After 1–2 years in the sea, adults return to their natal stream to breed. There is also a varying, but small, proportion of larger resident (non-migratory) trout that mature in the stream [23] and they typically occupy the deeper pools. Predators on juvenile trout are heron (*Ardea cinerea*), eel (*Anguilla anguilla*), larger resident brown trout, and mink (*Neovison vison*). We studied one-year-old (parr) brown trout of which most were expected to migrate after the end of the experiment in the late spring of 2009. On all occasions when fish were captured from the stream we used electro fishing from which the fish usually recover within a minute. This study was performed in accordance with Swedish animal welfare laws and was approved by the Gothenburg Ethical Committee for Animal research (113–2008). Electro-fishing permits were obtained from the County Administrative Board Västra Götaland and all field work was approved by the private land owners.

### Laboratory treatment

On 30-Apr-2008, a random sample of 200 brown trout were captured from the lower end of the stream and brought into the laboratory at the Department of Zoology in Gothenburg to become the starved treatment group. The following day, these fish were marked (PIT-tags), weighed and measured, and divided in equal numbers into four 100 liter tanks containing re-circulating freshwater at 10°C, coarse gravel, 5–10 rocks, and plastic plants. These fish weighed 6.9 g±0.2 SE and measured 86.2 mm±0.6, giving a condition factor (calculated as CF = 10^5^×W×L^−3^) of 1.05±0.01. Since fish in the starved treatment were presumably a random sample of the population when initially captured, we assumed that fish that remained in the stream, of which some were later to constitute the control treatment, were of the same average weight and length. Fish of the starvation treatment were food deprived for three weeks, because this has previously been shown to induce subsequent compensatory growth without compromising the health of the fish [18]. All fish were in good health upon release and there were no mortalities during this starvation period.

After a period of three weeks, on 18-May-2008, we captured 200 trout (to be control treatment) from the experimental section of the stream and brought them into the laboratory. The following day, these control fish were PIT-tagged, weighed and measured. In addition, starved fish were again weighed and measured. As expected, control fish that had grown in the stream were at this time point significantly larger (8.4 g±0.2 and 92.2 mm±0.7; Student's t-test: p<0.001 in both cases) than the starved fish (6.2 g±0.1 SE and 86.5 mm±0.7). Starved fish also had lower condition factor (0.92±0.04 SE) than control fish (1.04±0.04; p<0.001).

**Figure 1:**
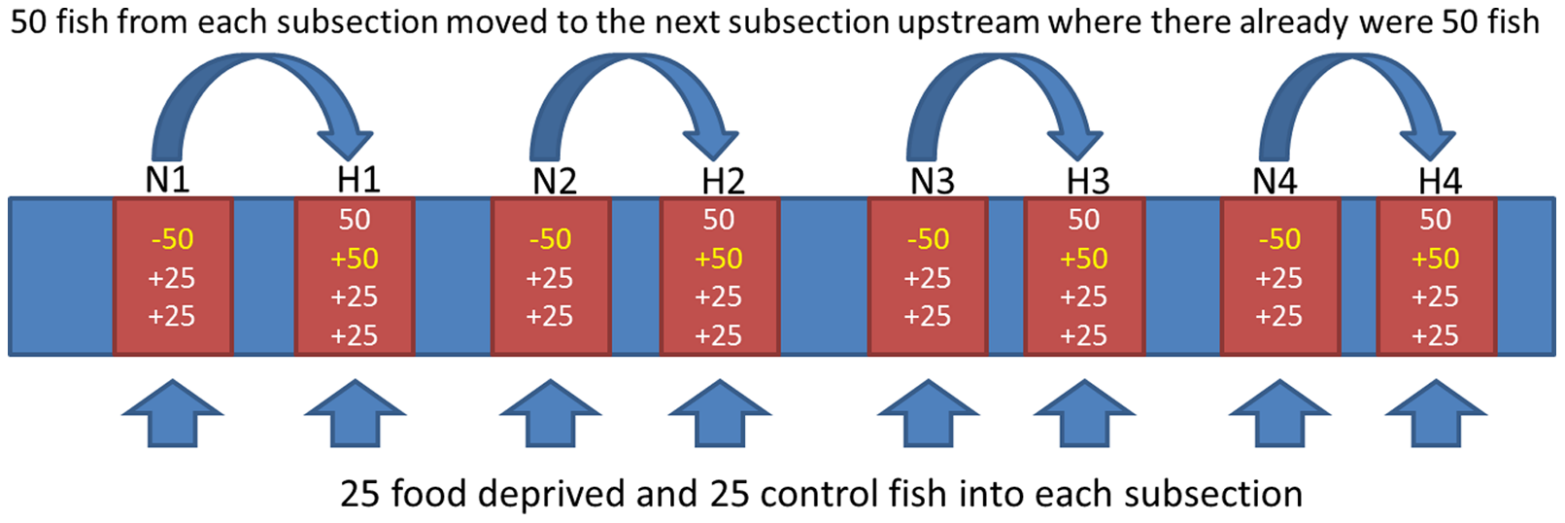
Schematic overview of the experimental stream showing buffer zones (blue) and experimental subsections (red), and the additions of fish into each subsection. Length of each experimental subsection was defined as containing 50 fish. In the natural density subsections (N), these fish (marked in yellow) were transferred to upstream sections (+50), and replaced with 25 treatment and 25 control fish from the laboratory, thus restoring the natural density of 50 individuals. High density subsections (H) had their initial 50 individuals, received 50 from the natural density subsections, and 25 treatment and 25 control fish from the laboratory, resulting in three times higher number than normal.

On 21-May-2008, the 200 control and 200 starved fish in the laboratory were split into eight containers, with 25 control and 25 treatment fish in each, and transported to the stream where fish were released into the center of each of eight subsections (Fig. 1) in the experimental stream.

### Stream experiment

On 20-May-2008, one day before returning the fish to the stream, we divided the stream section from which experimental (starved and control) fish had been obtained previously, into eight subsections separated by buffer zones (Fig. 1). The length of each subsection was determined by electro-fishing upstream until 50 individuals had been captured (range of subsections 25–55 m). Lengths of buffer zones (33–51 m) were determined by stream conditions so that they were expected to house about 50 individual trout each. Hence, each subsection contained at least 50 fish before the experiment started. From every other subsection, 50 fish were removed and replaced with the 50 experimental fish (25 starved and 25 control) thereby maintaining a natural density. These four subsections are referred to as natural density subsections (N1 to N4). The high density subsections (H1 to H4) were created by adding the 50 fish removed from sections N1-N4 and the experimental fish (25 starved and 25 control) thereby tripling the number of fish (50 existing, 50 added from downstream subsection, 25 starved, and 25 control fish added). This transfer of fish resulted in an average fish biomass of 1344 g±27 SD and density of 3.36±0.48 individuals per meter in H subsections and 355 g±32 SD and 1.4±0.50 individuals per m in N subsections. These numbers are approximate since not all fish were necessarily captured during this setup phase; fish would be able to emigrate out and into the subsections as well as to and from buffer sections where an unknown number of fish were present, and fish would grow over the experimental year possibly influencing functional density. In fact, during the study, densities in the subsections changed from an initial 2.4× in H subsections relative to N subsections upon release, to 1.25×, 1.15×, and 1.41× for the three recapture points (see below), respectively (including all fish captured in the stream).

Recaptures were made by electro-fishing the entire stream section, including buffer zones and experimental subsections, after 26 days (17/18-Jun-2008; first recapture after a period when fish in the system can grow rapidly [7] and when the potential for compensation would be great), 122 days (29–30 Sep 2008; second recapture after a longer period of slower growth and before adult spawners enter the stream), and 309 days (30-Mar-2008/01-Apr-2009; third recapture following the winter before most individuals initiate their downstream migration to the sea). Since this study was conducted under fully natural conditions, fish could freely move in the stream section and hence between subsections. We did not try to physically prevent movements with nets, because based on previous experience, nets are quickly flushed downstream. A previous experiment in this stream showed that trout normally move very little even at elevated densities [7].

### Statistical analysis

First we were interested in detecting whether fish compensated or not, then whether fish at the natural density were able to compensate more than those at the high density. The presence of compensatory growth in the starvation treatment relative to control treatment was assessed by analyzing the natural logarithm (ln) of absolute growth in weight and length (i.e. weight/length at a later recapture minus the weight/length at the preceding recapture, e.g. size at first recapture minus size at release), and changes in condition factor between recaptures using Restricted Maximum Likelihood (REML) in a Linear Mixed Model:
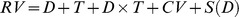
where RV  =  response variable (i.e. ln-transformed absolute weight change, ln-transformed absolute length change, change in condition factor), T  =  treatment (starved vs. control), D  =  density (high vs. natural), CV  =  covariate (weight at the beginning of a period for weight analyses, and length at the beginning of a period for analyses of length and condition factor), and S(D)  =  Density nested in random factor release subsection (H1–H4, N1–N4).

To detect a compensatory response, a starved fish would need to increase more in absolute size (weight and length) compared to a control fish of the same initial size after starvation treatment. When the random factor S(D) had p>0.25, data were pooled across subsections to increase power [24]. A high p-value for the random factor would imply that the release subsections do not significantly explain any differences in growth. For the last recapture, too few fish were recaptured from some subsections to include the random factor so this period was analysed on the pooled data only.

If a compensatory growth response was observed (i.e. significant treatment effect), a detailed analysis of the effect of density on compensatory growth was carried out on starved fish only. Given that we were interested only in the density effects on the compensatory response, we used only the starved fish for this analysis. It should be noted that density effects on natural growth has been documented previously [7]. The analysis of density on only starved fish therefore used the same model and selection procedure as described above, but excluded all treatment terms.

Mortality costs of compensation (i.e. recapture rate used as a proxy for survival), were assessed for each recapture time point using the pooled model (random factor removed) as above fitted to a binomial distribution with a logit-link function. To adjust for differences in “survival” prior to the onset of a period, the model-predicted recapture rate at the onset of a period was transformed and used as an offset variable in the analysis of the subsequent period. Hence, recapture rate in September and April was adjusted for the expected number of fish present in June and September, respectively, based on the recapture rate at that time. Because length at the start of the period was used as a covariate, only fish recaptured, which thus had a known length, could be included for assessment of survival to the September and April recapture time points.

Movements were variable and could not be made homogenous nor be made normally distributed. Instead, we grouped data into movements ≤20 m and >20 m as these would roughly represent fish that remained in their release subsection and fish that moved out of their release subsection, respectively. Movement data were then analysed using the same model as for recapture rate above.

By fishing the stream section twice on each recapture occasion we can estimate the probability of capturing a fish that is present [25]. First the population size in the section is estimated as n  =  c_1_^2^/(c_1_−c_2_) where n is the estimated number of fish in the section, c_1_ is the number of fish captured during the first fishing and c_2_ is the number of fish captured in the second fishing. Next, the proportion captured (c_1_+c_2_) is divided with the estimated population size n to give the probability of capturing a fish.For analyses of growth in weight and length, change in condition, and movements, only fish recaptured at subsequent electro-fishing occasions could be used. This resulted in 204, 100, and 47 fish being available for analyses for periods 1, 2, and 3, respectively.

Below, we focus on effects relevant for our hypotheses only, i.e. 1) did fish compensate, and if so 2) did this compensation differ depending on population density? Only the final statistics are provided. Hence, if the random factor subsection had P>0.25, only output from the pooled data is provided. Due to low numbers of fish recaptured in some stream subsections during the last period (Oct-Apr), all analyses on this last period focused on pooled data only. Complete statistical output for interactions, covariates, random factors, and model selection procedures are provided in the online supplementary material (Appendix S1).

## Results

Of the 400 fish released in May, 204 were recaptured in June, 141 in September, and 83 the following April. The estimated probability for capturing an experimental fish during the three recaptures was 94.1%, 87.4%, and 99.4%, respectively. These high recapture rates suggest that fish not recaptured where no longer in the stream section that was sampled but had either migrated or died. Of the tagged fish, 85 control (42.5%) and 47 starved (23.5%) fish were never recaptured.

### Weight growth

**Figure 2:**
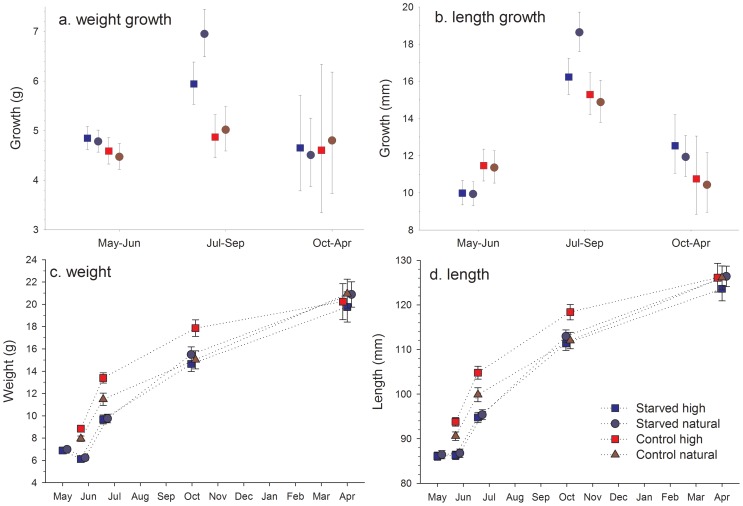
Absolute growth in weight (a) and length (b), and growth trajectories in weight (c) and length (d) in brown trout (*Salmo trutta*) during each of the three periods. Fish were starved (starved) or not (control) in the laboratory followed by release to nature at a natural (natural) or experimentally increased (high) density. Values in (a) and (b) are based on back-transformed estimated marginal means ± SE.

There was no significant difference in weight growth between starved and control fish during the first period (May-June; random factor retained with P = 0.13, treatment: F_1,__15.4_  = 0.94, P = 0.35). However, a clear compensatory growth response is observed for the second period (Jul-Sep) when starved fish grew more in weight than did control fish (random factor removed with P = 0.35, F_1,95_  = 11.8, P<0.001). Analysis of density effects, by including the starved fish only, showed that those fish at a natural density gained on average 17% more in weight than those fish at a higher density (F_1,63_  = 4.2, P = 0.045), indicating the presence of density-dependent compensatory growth during this period (Fig. 2a). There were no significant differences in weight growth between starved and control fish during the last period (Oct-Apr; F_1,42_  = 0.01, P = 0.91) which was clearly associated with an increased variation in growth among individuals (Fig. 2a). Final weight of the four groups were similar (overall average 20.5 g±5.9 SD; one-way ANOVA F_3,79_  = 0.2, P = 0.91) suggesting that a complete catch-up in weight had been achieved by the starved fish at both the high and natural density.

### Length growth

There was no significant difference in length growth between starved and control fish during the first period (random factor retained with P = 0.08, treatment: F_1,__13.6_  = 2.0, P = 0.18). For the second period (random factor removed with P = 0.36), starved fish grew on average more in length than control (F_1,95_  = 4.2, P = 0.043), indicating the presence of compensatory length growth. When looking at the starved fish only, those at a natural density grew on average 15% more in length than those at a high density (F_1,63_  = 3.9, P = 0.054), indicating the presence of density-dependent compensatory growth in length similar to that found for weight (Fig. 2b). There were no significant effects of treatment on length growth during the last period (F_1,42_  = 0.1, P = 0.33). Final length of the four groups were similar (average 125.6 mm±11.9 SD; F_3,79_  = 0.2, P = 0.87), suggesting that starved fish were eventually able to catch up in length.

### Condition factor

**Figure 3:**
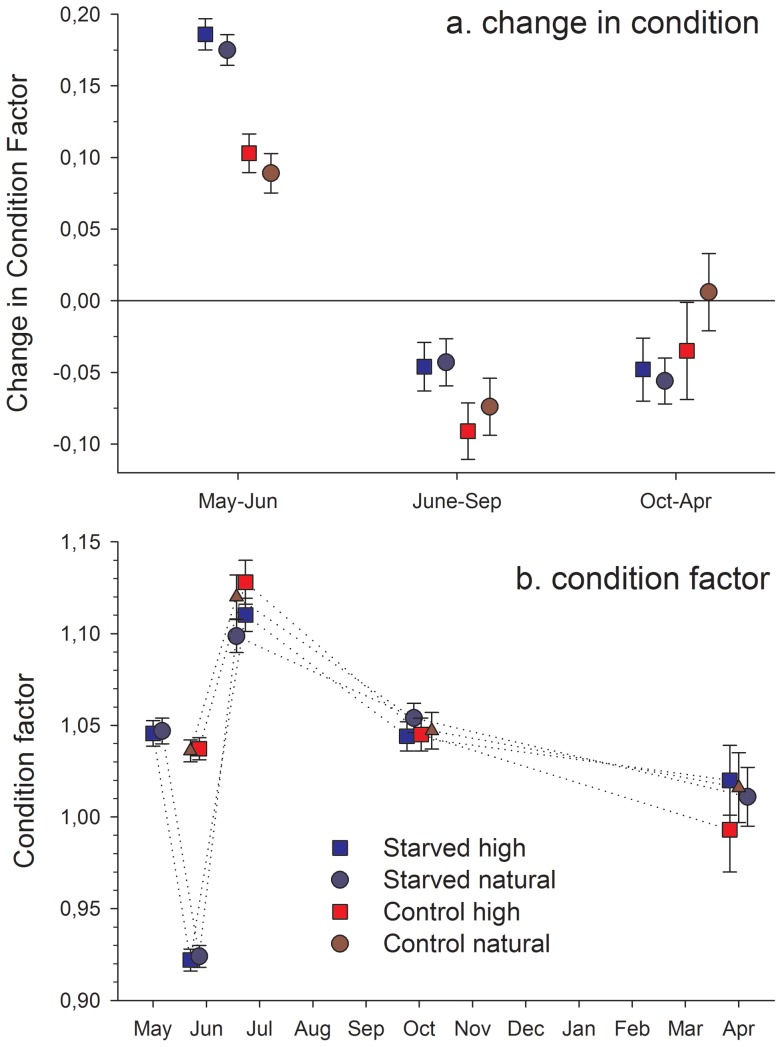
Change in condition factor (a) and condition factor (b) for brown trout (*Salmo trutta*) during each of the three periods. Fish were starved (starved) or not (control) in the laboratory followed by release to nature at a natural (natural) or experimentally increased (high) density. Values in (a) are based on back-transformed estimated marginal means ± SE.

For the first period (random factor removed with P = 0.35), starved fish increased more in condition than did control fish (F_1,199_  = 56.2, P<0.001), but density had no effect on condition in the starved fish (F_1, 127_  = 0.7, P = 0.42; Fig. 3). The effect on condition appears to arise from a relatively faster weight increase and slower length growth in starved fish compared to the control group (Fig. 2a and b). During the second period, starved fish lost less in condition than the fish in the control group (random factor removed with P = 0.26; F_1,95_  = 7.7, P = 0.007) probably because they started this period with a lower condition. Detailed analysis on starved fish revealed no effects of density on condition factor during the second period (F_1,63_  = 0.16, P = 0.69). There were no significant effects of starvation on change in condition during the last period (F_1,42_  = 2.27, P = 0.14). Final condition factor was the same among the groups (average 1.01 g±0.08 SD; F_3,79_  = 0.3, P = 0.83) matching the findings of no differences in weight and length.

### Recapture rate

**Figure 4:**
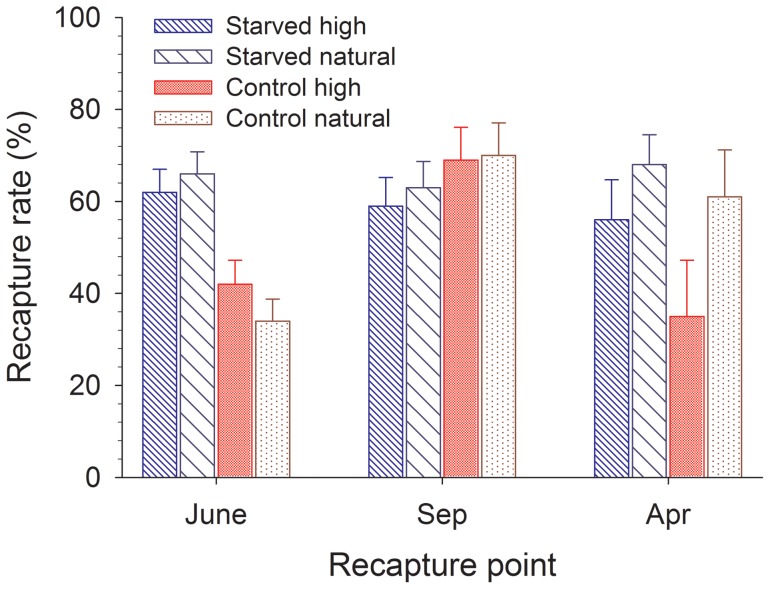
Recapture rate of brown trout (*Salmo trutta*) at each electro-fishing occasion adjusted for assumed survival based on recapture rate during the preceding period. Fish were starved (starved) or not (control) in the laboratory followed by release to nature at a natural (natural) or experimentally increased (high) density. Based on estimated marginal means ± SE.

At the first recapture, more starved fish were recaptured than fish from the control group in the experimental section (χ^2^  = 24.7, P<0.001), and analysis on starved fish only did not show any effects of density on recapture rate (χ^2^ = 0.4, P = 0.55; Fig. 4). This difference in recapture rate is likely due to differences in movement rather than survival (see below). At the second recapture, after adjusting for recapture rate during the first period, there were no differences among the fish due to treatment (χ^2^  = 1.4, P = 0.24). Recapture rates after the winter did not differ between starved and control fish (χ^2^  = 2.2, P = 0.14) but was higher in fish released into natural density subsection compared to high density subsections (χ^2^  = 4.0, P = 0.046). This finding indicates that long-term costs may be higher due to increased density effects relative to effects of starvation and/or compensatory growth.

### Movements

**Figure 5:**
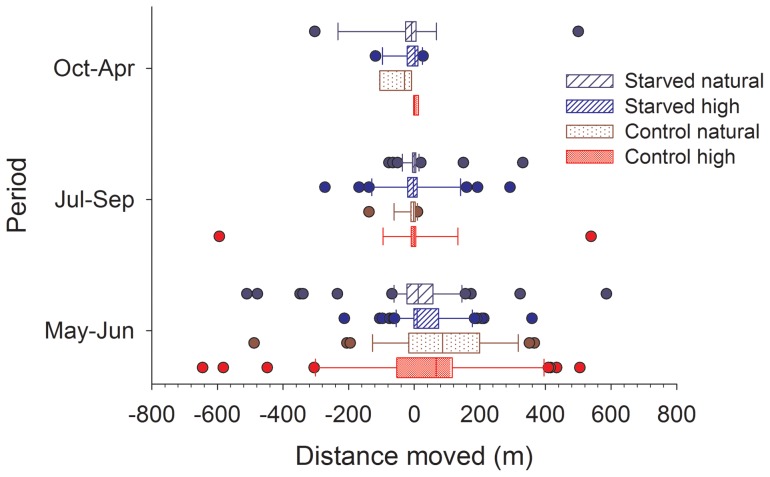
Movements of brown trout (*Salmo trutta*) during each of the three periods. Box-and-whisker plot showing median (vertical line inside box), 25 and 75 percentiles (edge of box), 10 and 90 percentiles (whiskers) and individual data points beyond (filled circle). Fish were starved (starved) or not (control) in the laboratory followed by release to nature at a natural (natural) or experimentally increased (high) density.

The number of fish in the control group that moved more than 20 m, were higher than the number of starved fish that moved more than 20 m during the first period (χ^2^  = 9.4, P = 0.002; Fig. 5), which may help explain the lower recapture rates in fish from the control group after this period of only one month (Fig. 4). Among the starved fish, individuals at the high density were more likely to move compared to those at the natural density (χ^2^  = 6.7, P = 0.009). During the second period, there was no effect of the starvation treatment (χ^2^  = 0.73, P = 0.39), but overall more fish moved at the high density compared to the low density (χ^2^  = 6.6, P = 0.010). During the last period, all fish from the control group at the high density had moved more than 20 m so this data could not be analyzed using the full model. Analysis of the starved fish did not indicate any effect of density on movement (χ^2^  = 0.31, P = 0.58).

## Discussion

We found that young brown trout, deprived of food for three weeks in the spring, showed compensatory growth in terms of weight and length over the summer, with a more pronounced response at a natural population density than at an experimentally increased density. This suggests that compensatory growth, just as normal growth in this species [7], is density-dependent. Previous studies, including those in our system, have found that fish first restore weight, when food is typically abundant and potential for growth is high [7], [8], [18], [19]. Indeed, even though we found no effect on weight or length growth during spring we did see a significant increase in condition factor in starved fish relative to fish in the control group, suggesting that starved fish first restored energy reserves (weight) before growing in structure (length), which seems to be the case for most fishes [13]. The somewhat delayed growth response observed in the present work may be due to the unusually low water levels in the spring (noted at the time of first recapture), which we believe also caused extensive movements, particularly in control fish, during this period. Low water levels may also have decreased the feeding ability by, for example, reducing the input of allochthonous energy to the stream (by a reduction in the stream surface area), in addition to reducing the available habitat for the fish, which may increase competition and aggression. The lack of differences in weight, length or condition factor among the four groups after the winter could be due to full compensation by starved fish with no effects of density, although we note that data at this point were highly variable and sample sizes lower than before the winter.

An unexpected finding in this study is the lower recapture rate of control fish relative to starved fish after the first period of only 26 days. It seems unlikely that the control fish, which were larger than the starved fish at this point, would suffer such high mortality during this short period. Rather than experiencing increased mortality, we believe that the control fish moved, to a greater extent than starved fish as suggested by our analysis on movements, to other sections of the stream as water levels dropped. This would also agree with previous work showing that brown trout choose deeper and slower-flowing water with increasing size [26]. Therefore, fish from the control group may have been more likely to emigrate out of the study section during this period in search for deeper pools. However, we also note that natural selection may act against large body-size increasing mortality and/or emigration at low water levels [27], [28]. With no differences in recapture rate after the summer, during which most of the compensation occurred, we have no indication that there were direct costs of compensation, such as increased predation often associated with rapid growth [29], [30].

In the present study, we did not find any delayed mortality cost of compensatory growth over the winter which is in agreement with a previous field study on brown trout using moderate feed restriction in the laboratory before return to the stream [17]. However, in a more recent study including groups with a feeding restriction comparable to the present study, compensatory growth did result in increased winter mortality [18]. Moreover, in the present study fish released at higher densities had lower apparent winter survival than those released at natural densities, which also suggests that three weeks of starvation per se did not compromise the survival of the fish. In contrast, in a previous field experiment on brown trout parr [7], density affected growth but not survival. These variable effects found in comparable stream model systems suggest that environmental variations can greatly influence the interactive effects of density, compensatory growth, and survival [31].

Movements during the first 26-day period altered the experimental density in each subsection as well as shifted the density experienced by individual experimental fish. Most fish probably experienced the high experimental density for only part of the initial four weeks after release, which suggests that our results are conservative. This is particularly interesting since, despite the limited compensation during this short initial period, we found compensatory growth over the summer. More controlled field studies will be required to evaluate the relative effects of the duration and magnitude of density fluctuations.

In most studies, the compensatory growth response in fish is achieved mainly by hyperphagia and to a lesser extent increased conversion efficiency and reduced metabolism [13]. This provides a link to the effects of density on compensation because a hyperphagic response is likely to increase competition over limited resources and/or increase social interactions resulting in increased energy expenditure [32]–[34]. As such, it will be more difficult to compensate at a high population density than at a lower density, keeping other factors constant. These effects may be particularly costly for the compensating fish, since they are smaller due to the growth restriction, compared to their non-starved conspecifics, and this may explain why the potential mortality effect (i.e. low recapture rate) of density (assuming that movement did not contribute to reduced recapture at this point) found in the present study has not been observed in normally growing (i.e. non-compensatory) fish [7].

In summary, this study demonstrates that compensatory growth in nature can be density-dependent just as normal routine growth. Thus, restoration of adaptive individual growth trajectories [18] through compensatory growth may be environment-dependent. This adds to the basic understanding of the regulatory mechanisms underlying compensatory growth, but we are far from having a complete picture of the factors eliciting compensatory growth, how it is regulated, and why. A more complete evolutionary and ecological understanding of compensatory growth requires both experimental manipulation and examination under a variety of natural settings.

## Supporting Information

Appendix S1 **Detailed statistical output.**
(DOCX)
