# Supplementary material for: Density-Dependent Compensatory Growth in Brown Trout (Salmo trutta) in Nature
Source: PLoS One. 2013 May 3;8(5):e63287. doi: 10.1371/journal.pone.0063287 (PMC3643939; doi:10.1371/journal.pone.0063287)
Supplement: Appendix S1 — Detailed statistical output. (DOCX) [file pone.0063287.s001.docx]

ONLINE SUPPLEMENTARY MATERIAL

Appendix S1: Detailed statistical output

Density-dependent compensatory growth in brown trout (Salmo trutta) in nature

L Fredrik Sundström, Rasmus Kaspersson, Joacim Näslund, Jörgen I Johnsson

LFS: Uppsala University, Department of Ecology and Genetics/Animal Ecology, Evolutionary Biology Centre, Norbyvägen 18D, SE-75236 Uppsala, Sweden.

e-mail: fredrik.sundstrom@ebc.uu.se, fred.sundstrom@gmail.com

Linear Mixed Model:

RV = T + D + T × D + CV + S(D)

where RV – response variable (i.e. ln-transformed absolute weight change, ln-transformed absolute length change, change in condition), T – treatment (starved vs. control), D – density (high vs. natural), CV – covariate (weight for weight analyses, and length for analyses of length and condition factor, S(D) – Density nested in random factor release subsection (H1-H4, L1-L4).

When the significance levels of the random factor S(D) p > 0.25, data were pooled across subsections and analyzed without this random factor according to Quinn and Keough (2002). If the factor treatment was significant (i.e. fish compensated), a detailed analysis was carried out on starved fish only (to assess if compensation differed between high and low density).

Analyses on recapture rates and movements used a Generalized Linear Model with binary response variable and a log-link function. Outputs are from Type III test of fixed effects. Model selection was as follows 1) if the random variable has P>0.25, re-run the analysis without the random variable, 2) if compensatory growth observed (factor treatment P < 0.05) then re-run the analysis using only starved fish to examine if density had an effect on the compensatory response.

**References in Appendix S1.**

Quinn G, Keough MJ. 2002. Experimental Design and Data Analysis for Biologists. Cambridge, UK: Cambridge University Press.

Table 1a. Statistical output for period 0-1.

|  |  | Weight change | | |  | Length change | | |  | Change in condition | | |
| --- | --- | --- | --- | --- | --- | --- | --- | --- | --- | --- | --- | --- |
| Factor |  | Df | F | P |  | Df | F | P |  | DF | F | P |
| T |  | 15.1 | 0.94 | 0.35 |  | 13.6 | 2.0 | 0.18 |  | 13.4 | 36.0 | < 0.001 |
| D |  | 12.7 | 0.90 | 0.77 |  | 12.8 | 0.01 | 0.92 |  | 12.0 | 0.84 | 0.38 |
| CV |  | 196 | 31 | < 0.001 |  | 194 | 10.8 | 0.001 |  | 198 | 3.4 | 0.067 |
| T × D |  | 12.8 | 0.01 | 0.92 |  | 12.9 | 0.0 | 0.99 |  | 12.1 | 0.03 | 0.87 |
| S(T) |  |  | Z=1.5 | 0.13 |  |  | Z=1.8 | 0.08 |  |  | Z=0.94 | 0.35 |

Table 1b. Statistical output for period 0-1 when data were pooled across subsections. Lower section on starved fish only.

|  |  | Weight |  |  | Length |  |  | Condition |  |
| --- | --- | --- | --- | --- | --- | --- | --- | --- | --- |
| Factor |  | NA |  |  | NA |  |  | F_1,199_ | P |
| T |  |  |  |  |  |  |  | 56.2 | < 0.001 |
| D |  |  |  |  |  |  |  | 1.6 | 0.20 |
| CV |  |  |  |  |  |  |  | 3.3 | 0.07 |
| T × D |  |  |  |  |  |  |  | 0.07 | 0.79 |
|  |  |  |  |  |  |  |  |  |  |
| Density effects |  | NA |  |  | NA |  |  | F_1,127_ | P |
| D |  |  |  |  |  |  |  | 0.66 | 0.42 |
| CV |  |  |  |  |  |  |  | 5.0 | 0.027 |

Table 2a. Statistical output for period 1-2. Lower section on starved fish only.

|  |  | Weight change | | | |  | Length change | | | |  | Change in condition | | |
| --- | --- | --- | --- | --- | --- | --- | --- | --- | --- | --- | --- | --- | --- | --- |
| Factor |  | Df | F |  | P |  | Df | F | P |  |  | Df | F | P |
| T |  | 14.2 | 8.0 |  | 0.013 |  | 13.2 | 2.7 | 0.12 |  |  | 9.9 | 4.0 | 0.074 |
| D |  | 12.7 | 1.2 |  | 0.29 |  | 12.0 | 0.42 | 0.53 |  |  | 9.2 | 0.8 | 0.40 |
| CV |  | 94 | 25.6 |  | < 0.001 |  | 94.6 | 0.02 | 0.89 |  |  | 93.1 | 4.4 | 0.038 |
| T × D |  | 12.6 | 0.9 |  | 0.37 |  | 11.9 | 2.1 | 0.18 |  |  | 9.1 | 0.4 | 0.55 |
| S(T) |  |  | Z=0.93 |  | 0.35 |  |  | Z=0.91 | 0.36 |  |  |  | Z=1.1 | 0.26 |

Table 2b. Statistical output for period 1-2 when data were pooled across release subsections. Lower section on starved fish only.

|  | Weight |  | Length |  | Condition |  |
| --- | --- | --- | --- | --- | --- | --- |
| Factor | F_1,95_ | P | F_1,95_ | P | F_1,95_ | P |
| T | 11.8 | < 0.001 | 4.22 | 0.043 | 7.7 | 0.007 |
| D | 1.3 | 0.26 | 0.27 | 0.61 | 2.0 | 0.16 |
| CV | 24.2 | < 0.001 | 0.05 | 0.83 | 4.3 | 0.040 |
| T × D | 1.1 | 0.31 | 2.77 | 0.099 | 0.9 | 0.34 |
|  |  |  |  |  |  |  |
| Density effects | F_1,63_ | P | F_1,63_ | Pp | F_1,63_ | P |
| D | 4.19 | 0.045 | 3.85 | 0.054 | 0.16 | 0.69 |
| CV | 9.55 | 0.003 | 1.52 | 0.22 | 2.92 | 0.09 |

Table 3. Statistical output for period 2-3 when data were pooled across release subsections.

|  | Weight |  |  | Length |  |  | Condition |  |
| --- | --- | --- | --- | --- | --- | --- | --- | --- |
| Factor | F_1,42_ | P |  | F_1,42_ | P |  | F_1,42_ | P |
| T | 0.01 | 0.91 |  | 0.96 | 0.33 |  | 2.27 | 0.14 |
| D | 0.00 | 0.98 |  | 0.07 | 0.79 |  | 0.43 | 0.52 |
| CV | 0.06 | 0.81 |  | 0.65 | 0.43 |  | 0.05 | 0.82 |
| T × D | 0.02 | 0.88 |  | 0.04 | 0.95 |  | 0.83 | 0.37 |

Table 4. Statistical output of recapture rates from Generalized Linear Model, binomial log-link, and likelihood ratio chi-square. Lower section on starved fish only.

|  | June |  |  | Sep |  |  | Apr |  |
| --- | --- | --- | --- | --- | --- | --- | --- | --- |
| Factor | Χ^2^ | P |  | Χ^2^ | P |  | Χ^2^ | P |
| T | 24.7 | <0.001 |  | 1.4 | 0.24 |  | 2.2 | 0.14 |
| D | 0.15 | 0.70 |  | 0.1 | 0.73 |  | 4.0 | 0.046 |
| CV | 3.3 | 0.070 |  | 0.0 | 0.93 |  | 2.0 | 0.16 |
| T × D | 1.3 | 0.25 |  | 0.1 | 0.75 |  | 0.5 | 0.50 |
|  |  |  |  |  |  |  |  |  |
| Density effects |  |  |  |  |  |  |  |  |
| D | 0.36 | 0.55 |  | NA |  |  | NA |  |
| CV | 5.63 | 0.018 |  |  |  |  |  |  |

Table 5. Statistical output of movements from Generalized Linear Model, binomial log-link, and likelihood ratio chi-square. Lower section on starved fish only.

|  | Period 0-1 |  |  | Period 1-2 |  |  | Period 2-3 |  |
| --- | --- | --- | --- | --- | --- | --- | --- | --- |
| Factor | Χ^2^ | P |  | Χ^2^ | P |  | Χ^2^ | P |
| T | 9.38 | 0.002 |  | 0.73 | 0.39 |  | NA |  |
| D | 2.45 | 0.12 |  | 6.60 | 0.010 |  |  |  |
| CV | 6.26 | 0.012 |  | 0.87 | 0.35 |  |  |  |
| T × D | 0.64 | 0.42 |  | 1.14 | 0.29 |  |  |  |
|  |  |  |  |  |  |  |  |  |
| Density effects |  |  |  |  |  |  |  |  |
| D | 6.74 | 0.009 |  | NA |  |  | 0.31 | 0.58 |
| CV | 3.52 | 0.061 |  |  |  |  | 0.40 | 0.53 |
